# Strong SOD2 expression and HPV-16/18 positivity are independent events in cervical cancer

**DOI:** 10.18632/oncotarget.24850

**Published:** 2018-04-24

**Authors:** Silvia Helena Rabelo-Santos, Lara Termini, Enrique Boccardo, Sophie Derchain, Adhemar Longatto-Filho, Maria Antonieta Andreoli, Maria Cecília Costa, Rafaella Almeida Lima Nunes, Liliana Aparecida Lucci Ângelo-Andrade, Luisa Lina Villa, Luiz Carlos Zeferino

**Affiliations:** ^1^ School of Pharmacy, Federal University of Goiás (UFG – Universidade Federal de Goiás), Goiás, Brazil; ^2^ Innovation in Cancer Laboratory, Center of Translational Research in Oncology, Cancer Institute of São Paulo (ICESP - Instituto do Câncer do Estado de São Paulo), Faculty of Medicine of University of São Paulo (FMUSP - Faculdade de Medicina da Universidade de São Paulo), São Paulo, São Paulo, Brazil; ^3^ Laboratory of Oncovirology, Department of Microbiology, Institute of Biomedical Sciences, University of São Paulo (USP - Universidade de São Paulo), São Paulo, São Paulo, Brazil; ^4^ Department of Obstetrics and Gynecololy, State University of Campinas (UNICAMP – Universidade Estadual de Campinas), Campinas, São Paulo, Brazil; ^5^ Laboratory of Medical Research, Faculty of Medicine of University of São Paulo (Faculdade de Medicina da Universidade de São Paulo-FMUSP), São Paulo, São Paulo, Brazil; ^6^ Institute of Life Sciences and Health, Faculty of Health Sciences, (ICVS), University of Minho, Braga, Portugal; ^7^ International Research Center, A.C. Camargo Cancer Center, São Paulo, São Paulo, Brazil; ^8^ Department of Pathology, State University of Campinas (UNICAMP – Universidade Estadual de Campinas), Campinas, São Paulo, Brazil; ^9^ Department of Radiology and Oncology, Faculty of Medicine of University of São Paulo (FMUSP - Faculdade de Medicina da Universidade de São Paulo-USP), São Paulo, São Paulo, Brazil

**Keywords:** human papillomavirus, cervical intraepithelial neoplasia, squamous cell carcinoma, adenocarcinoma, superoxide dismutase-2, Pathology

## Abstract

It is well known that persistent infection with high-risk HPV (hr-HPV), mostly HPV-16 and 18, is the main cause of cervical cancer development. Manganese superoxide dismutase (MnSOD or SOD2) are highly expressed in different neoplasia. The present study investigated SOD2 protein expression and the presence of hr-HPV types in 297 cervical samples including non-neoplastic tissue, cervical intraepithelial neoplasia grade 3 (CIN3), squamous cell carcinoma (SCC) and adenocarcinoma (ADC). Strong SOD2 expression was significantly higher in ADC (82%) than CIN3 (52%) or SCC (64%). There was no association between SOD2 expression and HPV 16 and/or 18 detection for every lesion analyzed. Binary Logist Regression revealed that strong SOD2 expression (OR: 27.50, 6.16-122.81) and HPV 16 and/or HPV 18 (OR: 12.67, 4.04-39.74) were independently more associated with CIN3 than non-neoplastic cervix. Strong SOD2 expression (OR: 3.30, 1.23-8.86) and HPV 16 and/or HPV 18 (OR: 3.51, 1.03-11.87) were independently more associated with ADC than SCC. Similar findings for SOD2 expression were observed by the Cochran Mantel-Haenszel test, controlling for HPV-16 and/or HPV 18. In conclusion, the expression of SOD2 was increased in CIN3 and SCC, and more increased in cervical ADC than in SCC. Strong SOD2 expression was statistically independent of the presence of HPV 16 and/or 18. These findings suggest that the mitochondrial antioxidant system and HPV infection could follow independent pathways in the carcinogenesis of cervical epithelium and in the differentiation to SCC or ADC of the cervix.

## INTRODUCTION

Cervical cancer is the second most common neoplasia in women, with estimated 500,000 cases occurring each year, globally resulting in >250,000 deaths [1–3]. Squamous cell carcinomas (SCC) represent 75 to 85% of all cases of cervical cancer, while adenocarcinomas (ADC) represent 11 to 25% of cases and adenosquamous carcinomas represent 2 to 3% of cases [4–6].

Virtually 100% of cervical cancers are associated with human papillomavirus (HPV) infection [7]. Twelve HPV types have been consistently classified as high–risk (hr-HPV) and all of them have been found in high-grade squamous intraepithelial lesions (HSIL), squamous cell carcinoma (SCC) and adenocarcinoma (ADC) of the cervix; other 13 HPV types have been classified as probably/possibly carcinogen and have been found in HSIL, and less frequently in invasive cervical cancer [8]. In cervical cancer, the most prevalent type is HPV-16, followed by HPV-18; together, these viruses account for around 70% of SCC and over 80% of ADC cases [9, 10].

In parallel, several cervical carcinoma-derived cell lines have been shown to be resistant to TNF anti-proliferative effect, suggesting that the acquisition of TNF-resistance may constitute an important step in HPV-mediated carcinogenesis. Analyzing the global transcription profile of normal and HPV-immortalized keratinocytes after TNFα treatment, using a microarray approach, manganese superoxide dismutase (MnSOD or SOD2) was identified as one of the differentially expressed genes in association with inflammatory response [11]. Superoxide dismutase proteins are highly expressed in different tumor types, and most of the current research efforts have been dedicated to understand their relation with the antioxidant system. Robust findings have shown that a subset of the SOD proteins is associated with cancer progression and metastatic phenotype [12–15]. These proteins are metalloenzymes that act in the cellular antioxidant system catalyzing the excess of superoxide anion to oxygen and hydrogen peroxide, which was thought to protect the cells from oxidative stress damage. Three distinct isoforms of SOD have been identified and characterized in mammals: copper-zinc superoxide dismutase (encoded by the *SOD1* gene), manganese superoxide dismutase (encoded by the *SOD2* gene) and extracellular superoxide dismutase (encoded by the *SOD3* gene). Moreover, SOD2 is found exclusively in the mitochondrial matrix and is an evolutionary conserved enzyme in a variety of organisms [14].

The pathway by which SOD2 expression might contribute to cancer development is not clear, but some researchers have suggested that, as a pro-oxidant protein, it promotes the accumulation of hydrogen peroxide, which can further lead to activation of various oncogenic pathways [16, 17]. Such oxidative metabolic product was recently demonstrated to inactivate regulatory proteins, shifting the paradigm from being a simple by-product to an important oncogenic regulator in carcinogenesis [16, 18]. In this context, one of the challenges is to understand under what circumstances hydrogen peroxide and, consequently, SOD2, plays a protective (antioxidant) or deleterious (pro-oxidant) role.

There is some evidence that oxidative stress and altered redox homeostasis might play a role as co-factors in cervical carcinogenesis [19, 20]. Termini et al. [20] evaluated SOD2 protein levels by immunohistochemistry in 331 cervical histological samples and observed that the frequency of SOD2-stained cells increases with cervical squamous disease severity, being particularly higher in ADC. Additionally, the SOD2 gene integrates a group of 11 genes identified as a signature of 33-fold increased risk for predicting pelvic lymph node metastasis in cervical carcinoma [21].

In summary, HPV infection has been considered a necessary event for cervical cancer occurrence, although not sufficient to trigger its development. Other factors not directly dependent of these viruses should act for the cells to achieve the malignant phenotype; oxidative stress could be one of them and the SOD2 protein expression a marker of this condition. The goal of the present study was to analyze if SOD2 protein expression level is associated with HPV infection, focusing on the 16 and 18 types, in ADC, SCC and cervical intraepithelial neoplasia 3 (CIN3) samples.

## RESULTS

HPV prevalence and type distribution according to the histopathological diagnosis are presented in Table 1. The overall prevalence of HPV was 83.6% in ADC (61 out of 73), 93.4% in SCC (57 out of 61) and 90.3% in CIN3 (93 out of 103). Most of the cases were positive for high-risk HPV types. The prevalence of HPV in women with non-neoplastic diagnosis (cervicitis and normal tissues) was 43.4% (26 out of 60). Women with non-neoplastic diagnosis that were included in the study due to suspicion of high-grade lesion were more likely to have detectable HPV-DNA, which probably justifies the prevalence of HPV in this group. Among the 26 women with non-neoplastic diagnosis that tested positive for HPV DNA, half was infected with low-risk HPV types (6, 11, 44 and 74). HPV-16 and/or HPV-18 were detected in 6.7% of non-neoplastic samples, 49.5% of cases of CIN3, 68.8% of cases of SCC and 69.8% of cases of ADC.

**Table 1:** Prevalence of HPV according to histological diagnosis

HPV detection	Diagnosis				All cervical samples n(%)
	Non-neoplastic	CIN3	SCC	ADC	
	n(%)	n(%)	n(%)	n(%)	
HPV-16 and/or 18^*^	4(6.7)	51(49.6)	42(68.8)	51(69.8)	148(49.8)
Other high-risk HPV ^*^	9(15.0)	42(40.7)	14(23.0)	4(5.4)	69(23.2)
Only low-risk HPV	13(21.6)	-	1(1.6)	6(8.3)	20(6.7)
HPV-positive	26(43.3)	93(90.3)	57(93.4)	61(83.6)	237(79.8)
HPV-negative	34 (56.7)	10 (9.7)	4 (6.6)	12 (16.4)	60 (20.2)
Total	60(100)	103(100)	61(100)	73(100)	297(100)

CIN3: cervical intraepithelial neoplasia grade 3; SCC: squamous cell carcinoma; ADC: adenocarcinoma. ^*^Single or multiple infections.

**Figure 1:**
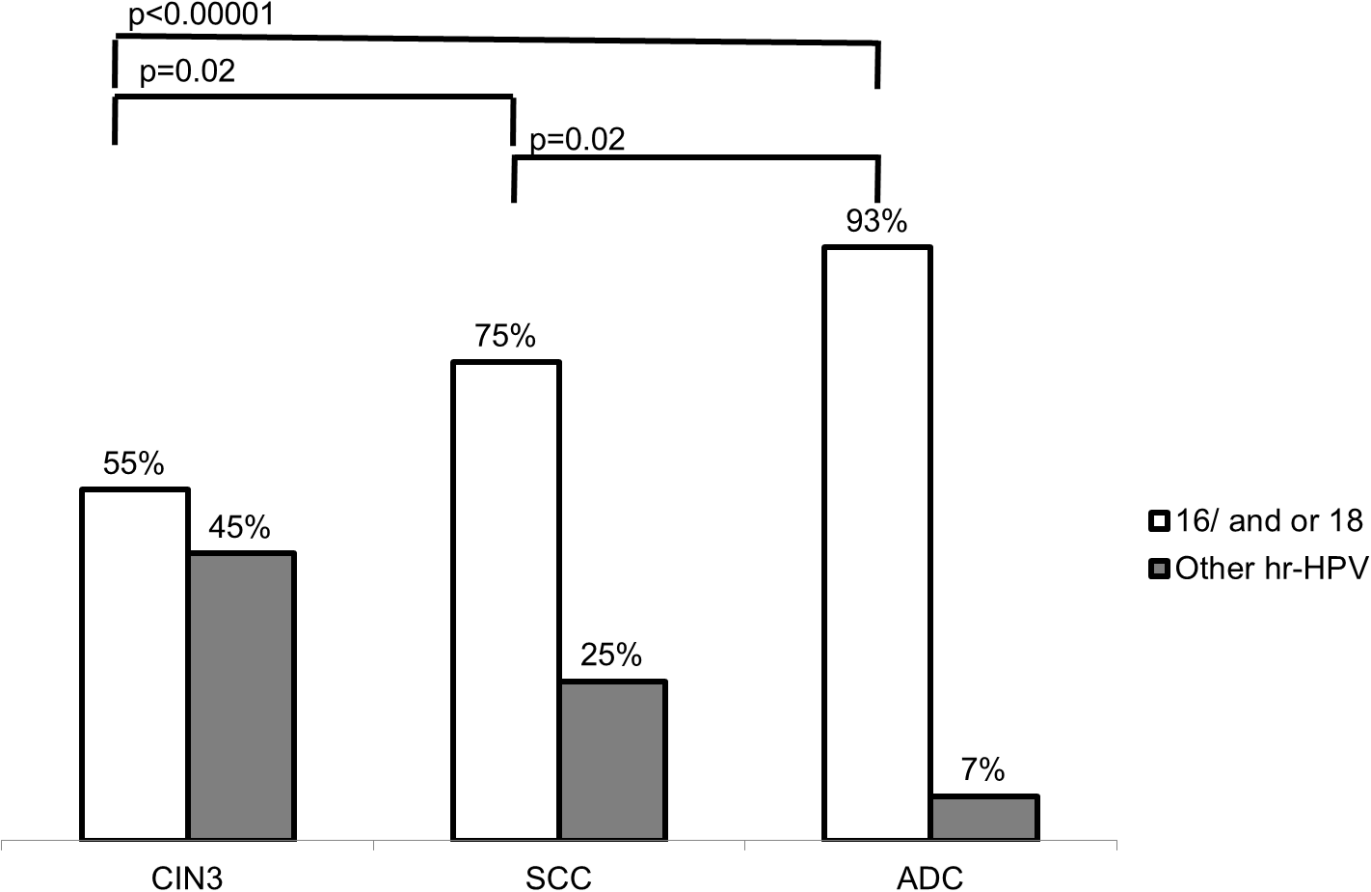
Association between high-risk HPV types and histological diagnosis of Cervical Intraepithelial Neoplasia (CIN3), Squamous Cells Carcinoma (SCC) and Adenocarcinoma (ADC) HPV-16 and/or 18 were compared with other high risk HPV types. Statistical analysis was performed by X^2^ test.

Figure 1 shows the association between HPV and the occurrence of cervical lesions, considering only the cases in which high-risk HPV types were detected. HPV-16/18 detection was significantly associated with ADC (*p* < 0.00001) and SCC (*p* = 0.02) when compared with CIN3. In addition, these two viral types were significantly more associated with ADC than SCC (*p* = 0.02).

**Figure 2:**
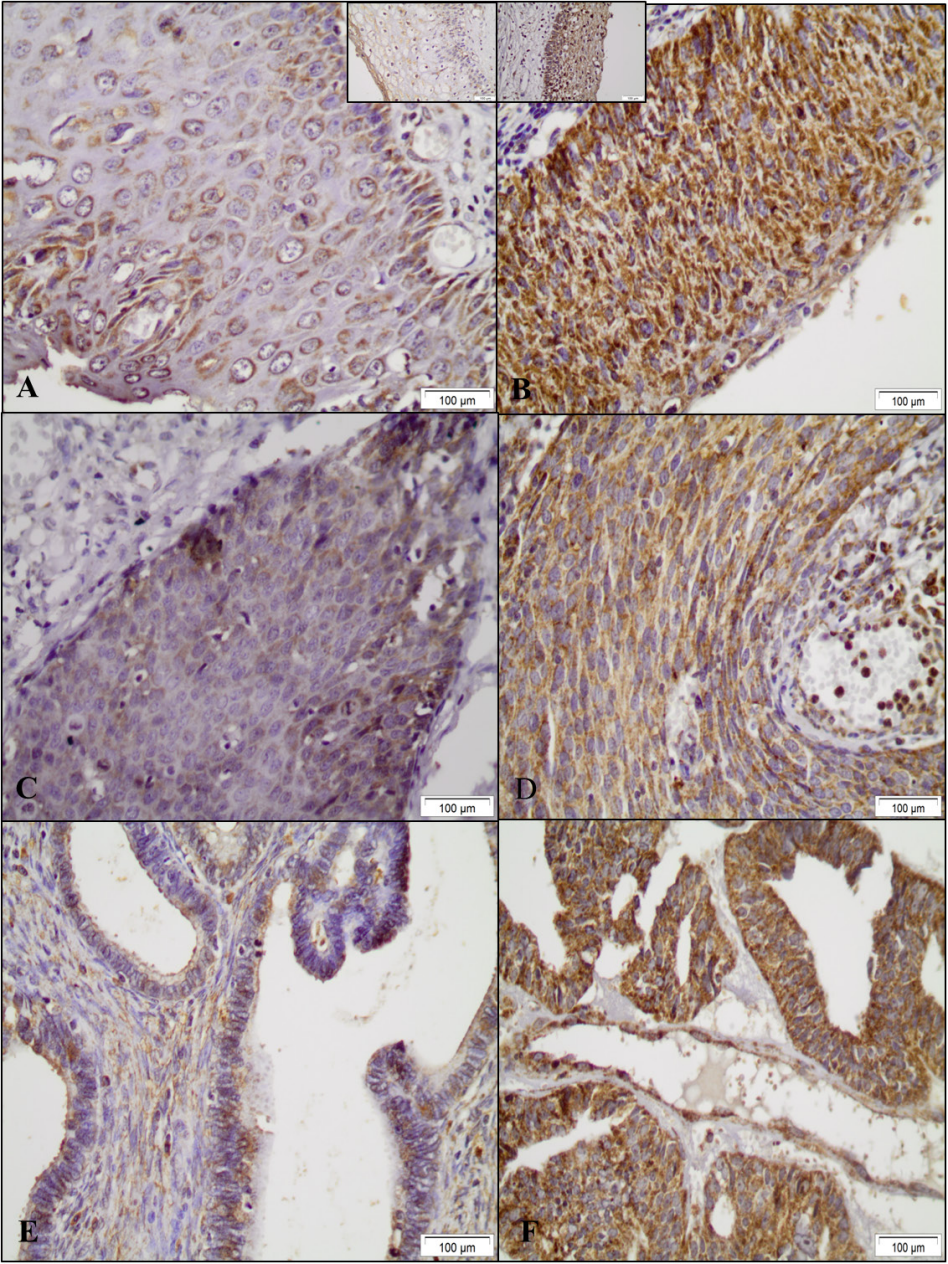
Representative examples of SOD2 immunohistochemical expression in cervical samples SOD2 expression levels in CIN3 **(A, B)**, SCC **(C, D)** and ADC **(E, F)** samples were determined by immunohistochemistry. Samples with less (A, C, E) or more (B, D, F) than 50% of the cells exhibiting SOD2 expression are presented. CIN3 - Cervical Intraepithelial Neoplasia grade 3, SCC - Squamous Cells Carcinoma ADC- Adenocarcinoma. (Bars: 100 μm). Non-neoplastic diagnoses with less or more than 50% of the cells exhibiting SOD2 expression are inset respectively in A and B.

The expression pattern of SOD2 protein was determined in the different histopathological samples using the non-neoplastic tissues as the control. As a general rule, SOD2 positive cells display a granular cytoplasmic staining pattern. Overall, a strong cytoplasmic SOD2 staining was observed in CIN3, SCC and ADC throughout the lesion, while no staining was observed in the stromal cells. However, in some cases, SOD2 expression was also observed in inflammatory cells infiltrating both epithelium and stroma. Representative examples of SOD2 staining in CIN3, SCC and ADC are presented in Figure 2.

**Figure 3:**
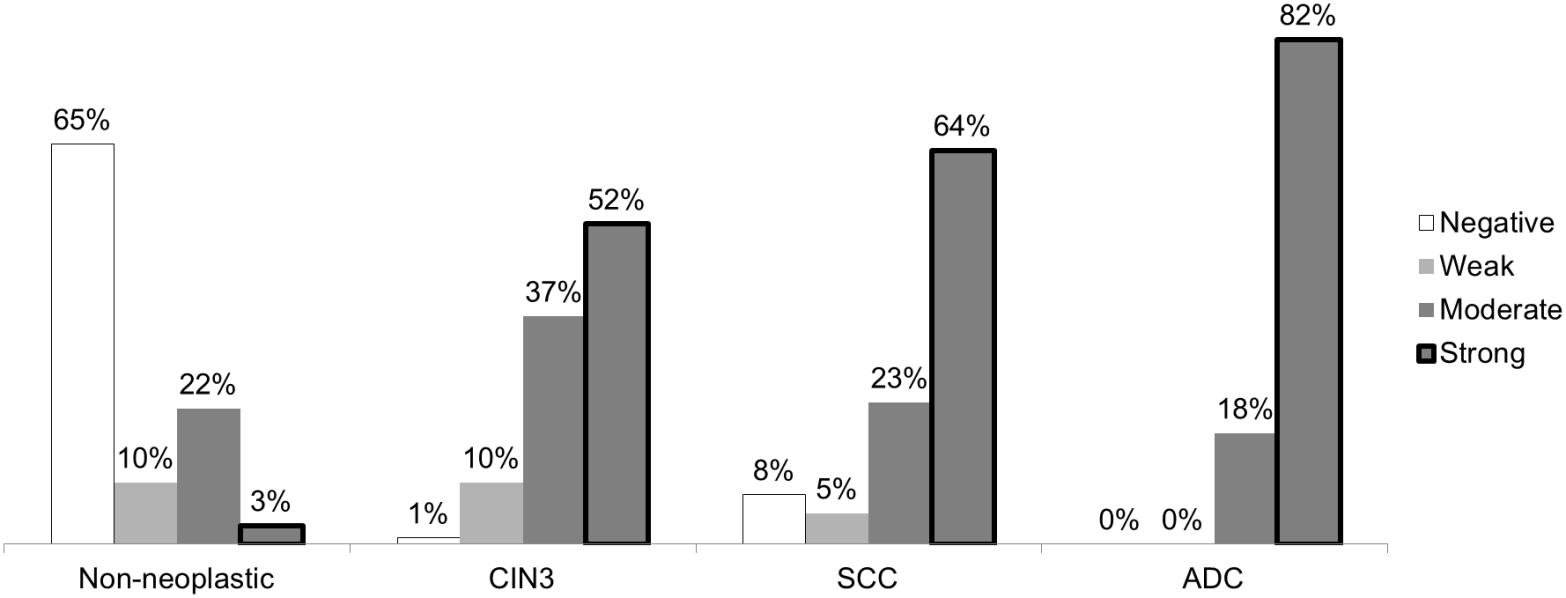
Strength of SOD2 staining according to histological diagnosis Expression was classified as negative or weak (<10% positivity), moderate (10–50% positivity) or strong (>50% positivity) in samples of cervical intraepithelial neoplasia (CIN3), squamous cells carcinoma (SCC) and adenocarcinoma (ADC).

**Figure 4:**
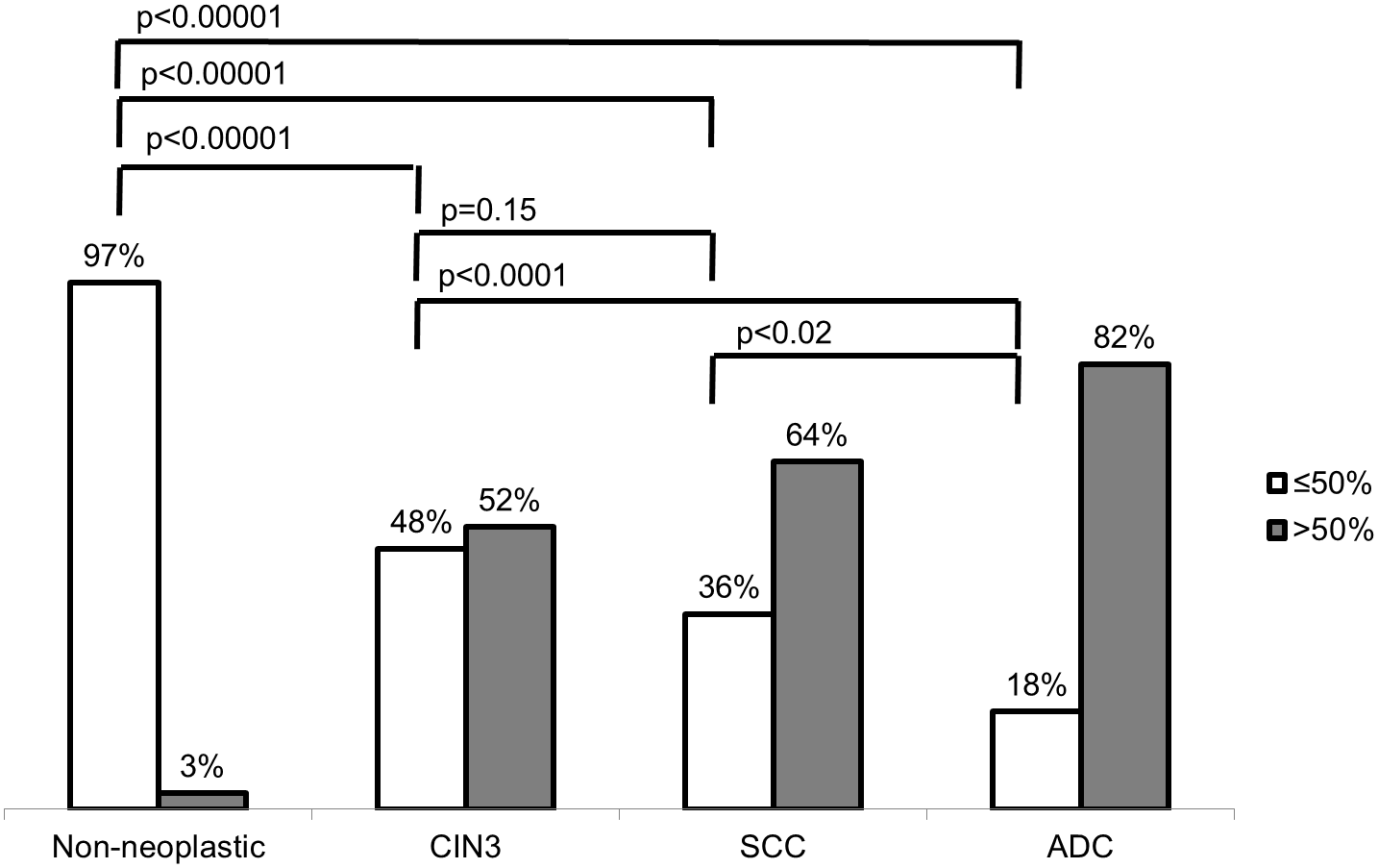
Comparative analysis of SOD2 expression in cervical samples SOD2 staining was determined as strong when positive in more than 50% of cells and weak or moderate when positive in less than 50% of cells. Strong SOD 2 expression was associated with cervical intraepithelial neoplasia and carcinoma when compared to non-neoplastic tissues. It was also associated with Adenocarcinoma (ADC) when compared to Squamous Cells Carcinoma (SCC) and Cervical Intraepithelial Neoplasia (CIN3). Statistical analysis was performed by the Fisher Exact test.

Most samples of non-neoplastic epithelia and normal glandular cells presented weak or negative staining for SOD2. Strong SOD2 expression (>50% positive cells) was observed in 3%, 52%, 64% and 82% of non-neoplastic samples, CIN3, SCC and ADC, respectively (Figure 3). Figure 4 shows that strong SOD2 expression was significantly higher in ADC, SCC and CIN3 (*p* < 0.00001) when compared with non-neoplastic tissues and was significantly higher in ADC than in CIN3 (*p* < 0.0001) and SCC (*p* = 0.02). No statistical difference was found between SCC and CIN3 (p=0.15).

Analyzing the SOD2 expression and HPV-16 and/or 18 for every histological diagnosis no association was observed between strong SOD2 expression and HPV-16/18 detection when compared with cervical samples positive for other high risk HPV types (Table 2).

**Table 2:** SOD2 expression in cervical samples with high-risk HPV types according to the diagnosis

Diagnosis	Hr-HPV type	Level of SOD2 expression^*^		p-value^**^
		Strong	Negative/weak/ moderate	
		n(%)	n(%)	
Non-neoplastic	HPV-1618	0(0)	4(100)	-
	Other hr-HPV	0(0)	9(100)	
CIN 3	HPV-16/18	27(56.2)	24(53.3)	0.84
	Other hr-HPV	21(43.8)	21(46.7)	
SCC	HPV-16/18	30(83.3)	12(60.0)	0.10
	Other hr-HPV	6(16.7)	8(40.0)	
ADC	HPV-16/18	44(91.7)	7(100%)	1.00
	Other hr-HPV	4(8.3%)	0(0%)	

CIN3: cervical intraepithelial neoplasia grade 3; SCC: squamous cell carcinoma; ADC: adenocarcinoma; hr-HPV: high-risk HPV. ^*^SOD2 expression was determined as strong when positive in 50% or more cells. ^**^Comparison between samples with HPV-16/18 and samples with other types of hr-HPV by Fischer Exact Test.

Strong SOD2 expression and HPV-16 and/or 18 positivity were identified as independent variables using binary logistic regression in an interdiagnosis comparison. Strong SOD2 expression (OR: 27.50, 95% CI: 6.16-122.81) and HPV-16 and/or HPV 18 (OR: 12.67, 95% CI: 4.04-39.74) were independently associated with CIN3 when compared with non-neoplastic diagnosis. Strong SOD2 expression (OR: 3.30, 95% CI: 1.23-8.86) and HPV-16/18 positivity (OR: 3.51, 95% CI: 1.03-11.87) were also independently associated with ADC when compared with SCC. Similar findings were observed for ADC when CIN3 was considered as reference. Strong SOD2 expression was not associated with SCC when compared with CIN3 (OR:1.54, 95% CI: 0.77-3.10). The Cochran Mantel-Haenszel test showed similar findings for strong SOD2 expression in all interdiagnosis comparisons, controlling for HPV-16 and/or HPV 18 (Table 3).

**Table 3:** SOD2 expression and HPV-16/18 detection in cervical neoplasias: an interdiagnosis comparison

Compared diagnoses	n	Binary Logistic Regression		Mantel Haenszel Test^*^
		Strong SOD2 expression^**^	HPV 16/18	Strong SOD2 expression^**^
		OR (95% CI)	OR (95% CI)	OR (95% CI)
CIN3 *vs* Non-neoplastic	106	27.50 (6.16-122.81)	12.67 (4.04-39.74)	13.95 (1.74-111.11)
SCC *vs* CIN3	149	1.54 (0.77-3.10)	2.35 (1.12-4.91)	1.54 (0.76-3.13)
ADC *vs* CIN3	148	3.86 (2.48-16.28)	4.06 (3.35-32.24)	5.85 (2.32-14.69)
ADC *vs* SCC	111	3.30 (1.23-8.86)	3.51 (1.03-11.87)	2.94 (1.09-7.89)

CIN3: Cervical Intraepithelial Neoplasia grade 3; SCC: Squamous Cell Carcinoma; ADC: Adenocarcinoma; hr-HPV: High-risk HPV.^*^Controlling for HPV 16/18. ^**^Positive in 50% or more cells.

## DISCUSSION

The role of SOD2 in cancer initiation and progression is not well understood. *In vitro* studies have demonstrated that overexpression of members of the SOD family correlates with increased cell differentiation, decreased cell growth and proliferation, and reversion of malignant phenotype [22]. Reduction of oxidative stress by increasing SOD2 levels might prevent DNA injury and consequently cancer development. Indeed, a protective role of SOD2 against tumor progression in transformed cell lines has been reported [23–25]. On the other hand, SOD2 activity might preclude the accumulation of H_2_O_2_, a component of reactive oxygen species (ROS), preventing programmed cell death or necrosis onset, thus favoring the malignant phenotype [12, 15, 26].

A recent review on SOD2 and carcinogenesis summarized that ROS largely originating in the mitochondria play essential roles in the metabolic and (epi) genetic reprogramming of cancer cell evolution towards more aggressive phenotypes. Therefore, there is likely a dichotomy where SOD2 can be considered a protective antioxidant reducing superoxide, as well as a pro-oxidant factor during cancer progression, with these effects depending on the accumulation and detoxification of H_2_O_2_ [18]. Nevertheless, the current understanding converges to the consensus that cancer cells exhibit a wide range of metabolic phenotypes that accumulate excessive ROS in relation to normal cells. Such ROS accumulation induces cellular damage that may promote cancer development as well as its metabolic phenotype [13-15, 27]. In fact, most of the studies about SOD2 expression and cancer have shown that overexpression of this enzyme is associated with the presence of metastases and poor prognosis in many malignancies, such as lymphomas [28, 29], glioblastoma [30], bladder [31], lung [32], colorectal [33], breast [34], penile [35], gastric [36], esophageal [37] and oral cancer [38].

In relation to cervical cancer and its precursor lesions, Termini et al. [20] examined the expression of SOD2 in a set of cervical samples, including low-grade squamous intraepithelial lesion (LSIL), HSIL, SCC and ADC. Those authors observed that HSIL and invasive cancers exhibited higher SOD2 levels than LSIL and, in most LSIL samples, they observed no or low SOD2 expression. On the other hand, a significant percentage of HSIL, SCC and ADC were included in the >50% stained cell category, exhibiting predominantly cytoplasmic granular deposits in all the layers of the epithelium. However that study did no mention about patterns of SOD2 expression and HPV infection for any circumstances.

Our study showed that the detection rate of HPV 16 and/or 18 increased with the squamous lesion severity, and this finding was expected [8]. Strong SOD2 expression was higher in CIN3 in relation to non neoplastic tissue, but in relation to SCC the difference was not statistically significant. This finding cannot be directly compared with those of Termini et al. [20] that showed little bit higher percentage of cases with strong SOD2 expression for HSIL than for LSIL, but the difference was not statistically significant. Even though HSIL classification includes CIN3, it also includes CIN2, which may have an LSIL phenotype [39].

Although the HPV types 16 and 18 are more typically prevalent in more severe cervical neoplastic lesions, such as strong SOD2 expression, no association was observed between strong SOD2 expression and HPV-16 and/or 18 in non-neoplastic tissue, CIN3, SCC or ADC samples. This information is new and suggests that SOD2 and HPV-16 and/or 18 infections could act in the carcinogenic process through different pathways, and not as co-factors. Additionally, this study showed that, controlling for the presence of HPV-16 and/or 18, strong SOD2 expression was significantly associated with CIN3 in relation to non-neoplastic cervical tissue, but there was no association between CIN3 and SCC. These findings are also new and reinforce the hypothesis that SOD2 could have a relevant and independent role in CIN3 development, but the data available do not support this assumption for the CIN3 progression to SCC.

Cervical ADC, in relation to SCC, occurs more frequently in younger and Caucasian women, and is generally associated with worse prognosis [40, 41]. The incidence of invasive cervical cancer has been decreasing in recent years. Nevertheless, cervical ADC (i.e., adenocarcinoma and adenosquamous carcinoma) stands out because its incidence among younger women has increased in more developed countries, even those with widespread screening programs [42]. In the United States, the proportion of ADC relative to SCC and to all cervical cancers doubled between 1973 and 1996, and the rate of ADC per population at risk also increased over this period [43]. In worldwide studies, HPV types 16 and 18 were present in, respectively, 56.8% and 11.6% of women with invasive SCC, and in 36.1% and 34.9% of women with ADC. However, cervical ADC and SCC differ not only with respect to the distribution of HPV types but also with respect to intratypic variants; non-European HPV-16 and HPV-18 variants are more commonly seen in ADC [44–46].

This study showed that SOD2 expression is especially higher in ADC in relation to other cervical samples, including SCC. Eighty-two percent of ADC and 64% of SCC cases revealed strong SOD2 expression, and no ADC cases showed negative or moderate expression. Termini et al. [20] described similar findings, with strong SOD2 expression in 66% of ADC and 40% of SCC. Our study also showed that, controlling for the presence of HPV-16 and/or 18, strong SOD2 expression was significantly associated with ADC in relation to CIN3 and SCC.

The pathway by which some types of HPV or variants could be associated with ADC or SCC is not clear. Also, no information was found in the literature about the SOD2 expression in non-neoplastic glandular epithelium of the cervix. This study indicates that the oxidative stress is higher in the ADC and the antioxidant system is strongly activated. At this point, it seems not possible to indicate how much the oxidative stress is an agent promoting the carcinogenesis or how much it is consequence of the cancer progression. The H_2_O_2_ produced in the mitochondria seems indispensable for hypoxic adaptation and energetic and/or metabolic homeostasis, and, therefore, it seems relevant to elucidate how much this process depends on and is controlled by SOD2. In this way, the better understanding of redox hubs in the mitochondria will likely lead to new and improved therapeutics of a number of diseases, including cancer [27, 47].

In conclusion, our results showed that the expression of SOD2 was increased in CIN3 and SCC, and more increased in cervical ADC than in SCC, and this pattern of SOD2 expression was statistically independent of the presence of HPV 16 and/or 18, the most prevalent types in the cervical cancer. These findings suggest that the mitochondrial antioxidant system and HPV infection could follow independent pathways in the carcinogenesis of cervical epithelium and in the differentiation to SCC or ADC of the cervix.

## MATERIALS AND METHODS

### Ethics statement

All tissue samples were originally collected for diagnostic purposes and were thoroughly anonymized before the use in this study. This study was approved by the State University of Campinas ethics committee (Protocol No. 04500146000-10). The institutional ethics committee waived the need for signed consent because this was a risk-free retrospective study and it was no longer possible to contact many of the enrolled women.

### Tissue samples

Case selection was based on pathology reports obtained from 2005 from women consecutively attended to at the Women’s Hospital Prof. Dr. José Aristodemo Pinotti, State University of Campinas, Brazil. Haematoxylin-eosin (H&E) paraffin-embedded sections were reviewed and the best representative samples were identified. Specimens were obtained by punch biopsies, large loop excisional cervical procedures, cones or hysterectomies. All women with non-neoplastic lesion and CIN3 had previous abnormal Pap test and they had undergone conization or loop excision of transformation zone of the cervix due to suspicion of squamous high-grade lesion.

Fixed and paraffin embedded tissues were cut into 3-μm sections and stained with H&E for histological analysis and selection of the appropriate tissue area for further investigations. This study included 297 cervical samples: 73 ADC, 61 SCC, 103 CIN3 and 60 non-neoplastic cervical tissue.

The sample size estimation was based on the data from the Termini et al. study of SOD2 expression in different stages of cervical neoplasia [20]. Those authors used two sided 95% confidence intervals and statistical power of 80%, and reported strong SOD2 cellular staining (positive in >50% of cells) in 40% of SCC cases and in 65.4% of cervical ADC cases; considering both SCC and ADC, that study used a total of 122 cases for Kelsey methods and 120 cases for Fleiss methods. In the present study, the total number of samples of these two tumor types was 134.

### HPV detection and genotyping

Cervical specimens with a confirmed histological diagnosis were analyzed for the presence of HPV DNA. Paraffin sections were systematically obtained from each block using a sandwich method (3-μm sections for H&E staining were taken immediately before and after the sections used for HPV DNA analysis). Total DNA was extracted using a proteinase K lysis procedure, as previously described [26]. The microtome blade was changed after each block was cut and all the surrounding area and apparatus were cleaned with xylene and ethanol after processing to avoid contamination between samples.

The Innogenetics (Gent, Belgium; now Fujibio) INNO-LiPA SPF-10 HPV Genotyping Extra assay was used according to the manufacturer’s instructions. This version of the assay allows the simultaneous and separate detection of 15 high-risk HPV types (HPV-16, -18, -31, -33, -35, -39, -45, -51, -52, -56, -58, -59, -66, -68, and -70) and 10 low-risk HPV types (HPV-6, -11, -34, -40, -42, -43, -44, -53, -54, and -74).

### Immunohistochemical SOD2 detection

After deparaffinization in xylene and rehydration in alcohol, antigen retrieval was performed by incubation in boiling 10mM citrate buffer, pH 6.0, for 20 minutes. Samples were incubated with an anti-SOD2 mouse polyclonal antibody (ab13533, Abcam, Cambridge, UK), at a 1:1500 dilution in 10% horse serum phosphate buffered saline solution for 18 hours at 4°C. Immunohistochemistry for SOD2 was performed according to Polymer Detection System (NovolinkTM Max Polymer detection Systems, Leica Biosystems, Newcastle, UK), as described elsewhere [20]. Sections derived from high-grade serous adenocarcinoma of the ovary were used as a positive control for SOD2 expression and were incubated in the absence or in the presence of anti-SOD2 antibody.

### Evaluation of SOD2 expression

The immunohistochemical assays were evaluated considering the percentage of stained epithelial cells. SOD2 expression was categorized based on cytoplasm positive reactions, as previously reported [20]; samples were classified as “negative/weak” (<10% stained cells), “moderate’ (10–50% stained cells) or “strong” (>50% stained cells). Immunohistochemical evaluation was performed independently and blindly by three observers (SHR-S, AL-F, LALA-A); discordant findings were discussed among them to achieve a consensus score.

### Statistical analysis

All statistical analyses were performed with the Statistical Package for Social Sciences (SPSS) 15.0 software. Samples diagnosed as non-neoplastic were used as the reference category for comparisons with CIN3, SCC and ADC. Furthermore, samples diagnosed as CIN3 and SCC were used as reference for ADC analysis. For the statistical analyses, the SOD2 expression was scored as less or equal to 50% of stained cells (negative to moderate expression) or more than 50% of stained cells (strong expression). The analysis of SOD2 expression and HPV-16 and/or 18 for every histological diagnosis was made using Fischer Exact Test.

The association between SOD2 expression and HPV types in CIN3, SCC and ADC samples, in an interdiagnosis comparison, was analyzed. For this purpose, binary logistic regression was used to estimate the probability of a binary response (different stages of the cervical neoplastic lesions) taking as predictor variables the SOD2 expression and HPV types. The Cochran Mantel-Haenszel test was used to confirm the strong SOD2 expression as a predictor variable for the different stages of neoplastic lesions, controlling for presence of HPV 16 and/or 18.
